# The Importance of the Management of Infectious Complications for Patients with Left Ventricular Assist Device

**DOI:** 10.3390/healthcare3030750

**Published:** 2015-08-26

**Authors:** Michinari Hieda, Makoto Sata, Takeshi Nakatani

**Affiliations:** 1 Departments of Transplantation, National Cerebral and Cardiovascular Center, Osaka 565-0873, Japan; E-Mail: tnakatan@res.ncvc.go.jp; 2 Pulmonology and Infection Control, National Cerebral and Cardiovascular Center, Osaka 565-0873, Japan; E-Mail: sata.makoto.hp@ncvc.go.jp

**Keywords:** Left Ventricular Assist Device, LVAD, drive-line infection, DLI, bloodstream infection, BSI

## Abstract

A left ventricular assist device (LVAD) therapy is the viable option for patients with advanced heart failure as a bridge to transplantation, bridge to recovery, or destination therapy. Although application of LVAD support has become a standard option, serious complications or adverse events related with LVAD remain a concern. LVAD-related infection including driveline infection (DLI) and bloodstream infection (BSI) is one of the serious clinical matters for LVAD patients, and especially BSI leads to the high incidence of mortality. The LVAD-related infections negatively impact patient’s quality of life. Therefore, control of infection is one of the primary goals of management in LVAD patients. Several efforts including early and appropriate intervention including antibiotics and wound care may contribute to avert the progress into BSI from localized DLI. Particularly, there are clinical secrets in how to use antibiotics and how to treat wound care in LVAD patients. The rational way of thinking for wound care will be introduced in this review.

## 1. Driveline Infection and Pump Pocket Infection as One of the Serious LVAD Complications

### 1.1. Driveline Infection

A left ventricular assist device (LVAD) therapy is the essential therapy option for patients with advanced heart failure and is also associated with serious adverse events including stroke, bleeding, right ventricular dysfunction, device malfunction, and infection [1]. The majority of LVAD-related infections occur around the driveline exit site, evolving with a stress or trauma to the binding between the skin and the driveline, and sometimes spreading into deeper inside [2]. If initial healing at driveline is inadequate, driveline infection (DLI) might be easily induced. LVAD-related infection including DLI and bloodstream infection (BSI) is a serious clinical matter for LVAD patients, and especially BSI leads to the high incidence of mortality [3]. DLI are thought to be the high risk of BSI, which is related to high mortality in LVAD patients. Indeed, BSI was a major cause of death in LVAD patients, and accounted for 41% of all deaths [4]. More than 60% of the BSI and DLI in LVAD patients were due to infection of Gram-positive cocci (GPC), such as *Staphylococcus species*, which are resident flora of the skin [5]. Therefore, wound care and antibiotic treatment are needed in LVAD patients who have GPC-positive culture in driveline exit site. Our previous report presented the importance of early and appropriate intervention including antibiotics and wound care for device-related infections to reduce the incidence of potentially fatal BSI [6]. Our strategies were not only prevention of infection during the perioperative period, but also subsequent strict wound site control including surveillance cultures, wound care, and proper use of antibiotics [6]. The LVAD-related infections negatively impact patient’s mortality and morbidity. Therefore, prevention and control of LVAD-related infections are essential managements in LVAD patients.

### 1.2. Pump Pocket Infection

Continuous DLI could develop to pump pocket infection [7]. Pump pocket infection should be suspected when the patients have positive culture of GPC, such as *Staphylococcus species*, for long time. Patients with pump pocket infection present with broad clinical signs of sepsis, including low grade fever like infectious endocarditis, and have symptom including mild to severe pain around the driveline or the implanted pump. Sprinter hemorrhage, Oslar nodule, and Janeway nodule, which are sometimes observed in patients with infectious endocarditis, might be a sign for pump pocket infection.

When pump pocket infection was suspected, two sets of blood cultures and CT scan should be performed for diagnosis as soon as possible. A handy-ultrasound device is very useful for seeking and following-up the fluid space around inside the driveline and the implanted pump. The measurement of fluid volume around the driveline and implanted pump by the handy-ultrasound device is helpful for assessment of pocket infection. The space around the pump might be occupied uninfected fluid especially early phase of post LVAD implantation. The Gallium-RI scan is useful for screening whether the fluid correction is inflammatory or not. The Gallium-RI scan reveal the local inflammation, and may be very useful method to know the area of infection.

The surgical drainage and adequate use of antibiotics are primary for the management of pump pocket infection [8]. The infectious tissue is taken out, and cultures for diagnose the bacteria or fungi are collected at the site. The pump pocket and the place around it are thoroughly cleaned by many lavage with saline. After surgical debridement, negative-pressure wound therapy (NPWT) is used to drain fluid and accelerate the healing of the pocket [9].

### 1.3. Effect of Post LVAD-Implantation Infections on Outcomes

The post LVAD-implantation infections have an impact on clinical outcomes in LVAD patients. In fact, there was a significant lower survival rate compared with patients without BSI. LVAD patients with BSI were significantly associated with increased in-hospital mortality [10].

In the early postoperative period, patients who had been BSI after LVAD-implantation had significantly higher rates of re-operation for bleeding, renal failure requiring dialysis, and multiple organ failure [10].

In long-term period after LVAD implantation, chronic DLI, following pump pocket infection, and BSI are associated with malnutrition, which related with poor outcome including deconditioning, risk of sepsis, and prolonged inflammatory response [11]. There was a significantly higher rate of thrombotic complications driven by neurological dysfunction and pump thrombosis of a device related infection [12].

DLI and BSI not only in early but also in late postoperative period are associated with mortality. Therefore, the early and appropriate interventions for infectious complications are crucially important.

## 2. The Criteria for Bloodstream and Surgical-Site Infection

For the early diagnosis of DLI and BSI, frequent observation of the driveline exit site and check of vital signs are extremely important. The criteria of infection consist of clinical signs accompanied by pain, fever, drainage, and/or leukocytosis. A positive culture from the suspected infectious site or blood is essential. Positive clinical signs of infection without positive culture also indicate the need for treatment. Elevated serum procalcitonin levels (>0.5 ng/mL), which have a sensitivity of 0.35 and specificity of 0.99 for the diagnosis of systemic infection [13], are helpful for diagnosis of BSI.

The criteria of the American College of Chest Physicians and Society of Critical Care Medicine (ACCP/SCCM) for treating bloodstream infections state that following 2 or more conditions should be found: (1) temperature > 38 °C or < 36 °C; (2) heart rate > 90 beats/min; (3) respiratory rate > 20 breaths/min or PaCO2 < 32 mmHg; and (4) white blood cell count greater than 12,000 cells/mm^3^, or less than 4000 cells/mm^3^, or 10% immature bands [14].

In addition, using the Hospital Infection Control Practices Advisory Committee (HICPAC) surgical-site infection criteria template, drive-line (and LVAD pocket) infections were defined as either: (1) purulent drainage from the drive-line exit site (or device pocket); (2) organisms isolated from an aseptically obtained culture of fluid or tissue from the driveline exit site (or device pocket); or (3) an abscess or other evidence of infection involving the drive-line tract (or device pocket) found on direct examination, during re-operation, or in a histopathologic or radiologic examination [15].

The patients meeting with those criteria should be coped with infectious complication as soon as possible.

## 3. Antibiotic Prophylaxis Protocol during LVAD Implantation

As an infectious control, the prevention during perioperative period is very important. Therefore, an example of antibiotic prophylaxis in our institute is given below. The antibiotic prophylaxis protocol includes teicoplanin (TEIC) (400 mg) 2 days before LVAD implantation and doripenem (1000 mg) within 1 h before skin incision [6]. Antibiotics are re-dosed based on their pharmacokinetic properties in the operation room [6]. Doripenem (1000 mg, 3 times daily) is administered until extubation after sterna closure, while TEIC (400 mg, once daily) and micafungin (150 mg, once daily) are administered until healing of the wound [6].

## 4. Routine Cultures of Driveline Exit Site

Routine cultures of driveline exit site were performed within 1 week from the day of LVAD implantation with additional cultures whenever there is clinical suspicion of infection, pain in the exit site, acute neutropenia or leukocytosis, or temperature <36 °C or >38 °C. Even if the appearance of driveline exit site is dry, or not infectious, it is important to keep the culture continued.

## 5. Biofilms

In LVAD patients, DLI is an inevitable infectious complication. Most of the offending bacterium involved with DLI, especially *staphylococcus species*, was considered to cause biofilms. As shown in previous reports, *staphylococcus species* is recognized as a biofilm producer [16] and accounts for a higher percentage of initial pathogen in driveline exit site [17]. The biofilms have dramatically increased resistance to antibiotics [18]. The bactericidal activity of cefazolin (CEZ) against *S. epidermidis* after formation of the biofilms decreased more markedly than that before formation [19]. Therefore, antibiotics and wound care should precede the emergence of the biofilm.

## 6. Wound Care

Careful sterile management of the driveline exit site is very important. The driveline exit sites receive sterile cleansing with diluted hydrogen peroxide and placement of an antimicrobial occlusive dressing for wound care, with dressing changes performed 2–3 times per day [6]. It is very essential to keep dry at the driveline exit site. If DLI has progressed with moist granulation tissue at the percutaneous insertion site, cauterization with silver nitrate is done to make it regress. Surgical debridement is undergone for relatively large DLI. After surgical debridement, negative-pressure wound therapy (NPWT) was used to accelerate the healing of the wound if necessary. NPWT is a therapeutic technique using a continuous vacuum dressing to prompt healing the wounds, and has been reported to be useful for the treatment of mediastinitis after surgery [20,21]. Since LVAD patients also have several risk factors for bleeding, including anticoagulation therapy and wound infection, it is necessary for the LVAD patients to pay attention to bleeding. Generally, NPWT will continue until the culture of the driveline exit site turns out to be negative.

## 7. Antibiotics

All DLI are treated with systemic antibiotics including CEZ, vancomycin (VCM), TEIC or linezolid (LZD) that cover *stapylococcus* as soon as possible. In a case with continuous infection of DLI over 1-month, antibiotics rotation should be conducted for adequate dosing periods. CEZ is a primarily selected antibiotics for DLI treatment, while VCM, LZD or TEIC are administered if once methicillin-resistant Staphylococcus aureus (MRSA) has been detected in the nasal cavity at preoperative period, or BSI is suspected. Early de-escalation of antimicrobials is performed based upon the results of Gram staining of exit site sample and/or blood culture. Antibiotics are dosed according to renal function when appropriate.

As a very important strategy for preventing BSI, antibiotics rotation has been widely adopted in a field of serious infection. Antibiotics rotation is the scheduled rotation of one or more different classes of antibiotics. The purpose of antibiotics rotation is to decline antimicrobial resistance through successive and prospective alterations in antibiotic selection pressures, preventing selection of specific resistance properties [22]. Although antibiotic rotation for GPC-positive DLI is conducted to prevent DLI leading to BSI, the details of optimal antibiotics rotation, such as rotation order and duration of each rotation, have not been clarified. Also, further study is needed to respond to the question “which antibiotics should be used as initial administration of antibiotics rotation for GPC-positive device-related infection?”

How to select the initial antibiotics for LVAD infection? From our experience in 22 LVAD patients who had GPC-positive in driveline exit site within 12-month after LVAD implantation from our previously reported cases [6], CEZ and VCM were administered as initial antibiotics to 15 patients and the other 7 patients, respectively. The incidence of DLI and BSI were examined between the two groups (follow-up period, 493 ± 264 days). Over 80% patients suffered from DLI within 12 months after LVAD implantation, there was no difference with the effect of initial antibiotics between CEZ and VCM group in the patients with culture-positive of GPC in driveline exit site for DLI and BSI within 1-year. *Methicillin sensitive Staphylococcus aureus* (MSSA) was detected from the around driveline of 11 patients, *S. epidermidis* from 3 patients, and *E.coli* from 1 patient of CEZ group first culture. MRSA was detected from the first culture around driveline of 2 patients, MSSA from 2 patients, *S. epidermidis* from 2 patients, and *K. pneumonia* from 1 patient of VCM group. The rate of initial MRSA detection in driveline in the patients was 9.1% (*n* = 2) in total of DLI. Although DLI were common infectious complication in the LVAD patients, BSI developed in only 13.3% of CEZ and 14.3% of VCM patients (log-rank test; *p* = 0.80). Bacterial organisms cultured from blood samples were found to be *P. aureginosa* (*n* = 1) and *S. bovis* (*n* = 1) in CEZ group and *S. epidermidis* (*n* = 1) in VCM group. These results suggest that CEZ might be acceptable as initial antibiotics in the LVAD patients with culture-positive of GPC in driveline exit site for DLI and BSI, if MRSA rate in the ward is low.

## 8. Conclusions

LVAD-related infections including driveline infection by GPC, especially *staphylococci species*, are common in LVAD patients. The driveline exit site is thought to be the main gate of entry for bacterium. Not only prevention of infection during the perioperative period but also the rigorous wound care including surveillance cultures, frequent sterilization, and proper use of antibiotics are essential. The early and appropriate intervention to wound care of driveline is crucially important to prevent fatal BSI induced by DLI.
